# Effectiveness of a Prevention Program for Gender-Based Intimate Partner Violence at a Colombian Primary School

**DOI:** 10.3389/fpsyg.2019.03012

**Published:** 2020-01-21

**Authors:** Anni Marcela Garzón Segura, Rodrigo J. Carcedo González

**Affiliations:** ^1^Faculty Social Sciences and Humanities, Andean Region University Foundation, Bogotá, Colombia; ^2^Department of Developmental and Educational Psychology, Faculty of Psychology, University of Salamanca, Salamanca, Spain

**Keywords:** intimate partner violence, prevention, middle childhood, primary education, gender stereotypes, socio-emotional skills

## Abstract

Intimate partner violence, particularly against women, is widely studied owing to its high rates, based on transnational data. Colombia, where this form of violence is considerably common, is no exception, and such violence is occurring more and more often in increasingly younger couples (10−14 years old). Further, risk factors such as wide acceptance, the justification of intimate partner violence, extremely rigid traditional gender roles, and poor socio-emotional skills play a crucial role. In accordance with this reality, a gender-based intimate partner violence prevention program was designed, implemented, and evaluated for primary school children in Colombia based on a review of successful preventive programs and an identification of the main predictors of intimate partner violence. The program was evaluated using a quantitative study with a quasi-experimental design that included an experimental and a control group. In total, 344 participants were involved in the study: 195 boys (56.7%) and 149 girls (43.4%) from the second and third grades of a primary school (average age: 7.8 years) at a Colombian educational institution. The experimental group consisted of 200 participants and the control group of 144 participants. The program’s effectiveness was evaluated by measuring three groups of variables (gender stereotypes, the acceptance of violence, and socio-emotional skills) using reliable scales. To analyze the program’s effectiveness, mixed ANOVAs with a within-subjects factor (when the group was measured), two between-subjects factors (group and gender), and a covariate (age) were used. The results showed that the participants in the experimental group had lower scores in gender stereotypes, acceptance of peer aggression, and acceptance of physical violence against women compared to the control group. Conversely, they had higher scores in affective empathy after the intervention; both groups showed no significant differences before the intervention. This program is highly relevant because it has proven to have a positive impact on the participants and is innovative due to the lack of preventive programs that have been implemented in primary education and evaluated within the Colombian context.

## Introduction

Intimate partner violence, particularly against women, is a notably common problem across several countries (Pan American Health Organization [PAHO], 2003). In Colombia, specifically, multiple factors contribute to intimate partner violence: the country suffers high rates of intimate partner violence in a variety of forms, particularly against women (according to the National Institute of Legal Medicine and Forensic Sciences, 2019), with 49,669 cases of intimate partner violence reported in 2018, of which 86% were against women.

Similarly, intimate partner violence is occurring in increasingly younger couples and in those who live separately. According to the National Institute of Legal Medicine and Forensic Sciences (2019), cases have been reported in couples with ages ranging from 10 to 14 years old (95 cases in 2016, 89 cases in 2017, and 73 cases in 2018) and from 15 to 17 years old (1,397 in 2016, 1,342 cases in 2017, and 1,217 in 2018), and primarily among domestic partnerships and single women (45.6 and 45.5%, respectively). In relation to this, romantic relationships begin at an early age in Colombia (10−14 years old), leading to teenage pregnancies and an increased risk of experiencing intimate partner violence during adulthood (Observatory on Gender Affairs, 2012; Economic Commission for Latin America and the Caribbean, 2015; National Administrative Department of Statistics, 2016).

### The Current Situation Regarding the Prevention of Gender-Based Intimate Partner Violence in Colombia

Currently, according to the Pan American Health Organization [PAHO] (2014), the only strategy whose scientific evidence demonstrates its effectiveness in preventing intimate partner violence is the implementation of school programs on the prevention of violence in relationships. Notwithstanding, the lack of programs aimed at preventing intimate partner violence in the educational environment plays a crucial role. In Colombia, only two programs aimed at implementing and evaluating the prevention of intimate partner violence in adolescence were found: the Martínez (2014) program and the Constructive Romantic Relationships Program (RRC, for its Spanish acronym), by Gómez (2014). Both programs proved to be effective in preventing partner violence in Colombian adolescents. This highlights the educational context as an enabling environment which has a bearing on the risk factors that cause violence.

However, taking into account that the mean age at which a romantic relationship begins in Colombia is younger than in other countries (Economic Commission for Latin America and the Caribbean, 2015) and the existence of reports of partner violence at 10–14 years old (National Institute of Legal Medicine and Forensic Sciences, 2019), there is a clear need to initiate preventive interventions before the onset of adolescence. In this regard, only one program focused on the prevention of gender violence was aimed at primary school students [the *Machismo no es destino* program (Fernández and Ayllón, 2014)]. While it should be noted that this program was proposed as a product of previous research evidencing sexist beliefs and tracking the presence of negative male models on Mexican boys and girls of 8 to 13 years of age (Vargas and Fernández, 2011; Bailón et al., 2013), unfortunately no evaluation of its efficacy was conducted.

Conversely, there is significant demand for the development of preadolescent intimate partner violence prevention programs (Gorrotxategi and de Haro, 1999; Segato, 2003; Alonso and Castellanos, 2006; UN Agency for Refugees – UNHCR, 2008; Fernández and Ayllón, 2014; Valle, 2015). After conducting a comprehensive review of relevant studies, no specific prevention program for primary school students has been found at the international level. All interventions were aimed at adolescents and young adults (e.g., Jaycox et al., 2006; Schwartz et al., 2006; Wolfe et al., 2009), and yet none at primary school students. Some programs were focused on gender inequality and gender roles at primary education, however gender or partner violence were not a central aspects of the program (e.g., Rainey and Rust, 1999). As a consequence, a new intervention had to be developed. The question at this point was how to select the contents of an effective program.

### The Development of a Program for the Prevention of Partner Violence in Primary Education

In order to develop a new intervention, four sources were considered of paramount importance: (1) developmental determinants, present before the age of 10, of partner violence in adolescence and/or adulthood; (2) determinants of partner violence in adolescence, the next and most proximal developmental stage; (3) contents of effective partner violence prevention programs in adolescence; and (4) theoretical models which explain partner violence.

With respect to the developmental determinants of partner violence in adolescence and/or adulthood present before age 10, the systematic review of 25 longitudinal studies conducted by Costa et al. (2015) has found as predictors of domestic violence (defined as violent acts between adult intimate partners, and can include physical, sexual, emotional and/or psychological abuse) perpetration and/or victimization related to child and adolescent abuse experiences (e.g., substantiated physical abuse before age six –Linder and Collins, 2005–), family of origin risks (e.g., weak attachment to parents and poor quality of family relationships –Magdol et al., 1998–), and behavioral problems (aggressive and antisocial behavior –Magdol et al., 1998–), and less consistently, sociodemographic risks (e.g., low socio-economic status in the family of origin). As for adolescence, the same aspects were found to be associated with domestic violence.

Likewise Farrington et al. (2017) conducted a review of 42 systematic reviews and meta-analyses (16 of them specifically focused on dating and partner violence), identifying the following risk factors for intimate partner violence: masculine gender-role stress (Baugher and Gazmararian, 2015), child abuse (Fry et al., 2012), peer risk factors (Garthe et al., 2017), childhood experiences of violence (Smith-Marek et al., 2015), economic stress, male dominance, male privilege (West, 2016), aggressive/antisocial peer behavior and dating violence (Garthe et al., 2017), and in general terms for the explanation of violence they found child abuse and the low empathy as significant individual risk factors.

Both reviews with different age groups, which also coincide with the determinants of violence among intimate adult partners, highlight the presence of differing experiences of violence or aggression, the acceptance of gender roles and gender stereotypes, and socioemotional competence like empathy as determinants of partner violence. With regard to experiences of violence and/or aggression, Colombia is one of the countries with the highest level of violence at a cultural level which has also been linked to increased levels of violence in other spheres such as romantic relationships (Noe and Rieckmann, 2013). One of the possible consequences of being in contact with violence is its normalization. A partner violence prevention program in primary education should therefore address attitudes toward partner violence because it has been identified as a key factor for primary prevention due to the significant relationship it has with aggressive partner behaviors among adolescents and young adults (Slep et al., 2001; Wolfe et al., 2004; Taylor and Mouzos, 2006; Próspero, 2007; Sears et al., 2007; Machado et al., 2009; Abramsky et al., 2011; Muñoz-Rivas et al., 2011). Given that risks have also been found among adolescent peer groups as significant predictors of intimate partner violence, addressing attitudes toward peer violence should also be incorporated into a preventive program.

Previous research also suggests that there are gender-related risk factors associated with intimate partner violence; in this way, gender roles and stereotypes are accepted, as well as a rigidity in the definitions of masculinity and femininity and a notion of masculinity based on authority, domination, honor and aggression (Foshee et al., 1998, 2004; Mahlstedt and Welsh, 2005; Aumman, 2006; Ferrer et al., 2006; Sears et al., 2007; González-Ortega et al., 2008; White and Smith, 2011; Shen et al., 2012); at the sociocultural level there is gender inequality, the sexual division of power and labor (Heise et al., 1999), socialization in patriarchal cultural norms (Dutton and Golant, 1997; Turinetto and Vicente, 2008) and a lack of visible female role models (Echeburúa and Fernández-Montalvo, 1998; Aumman, 2006). Thus, gender roles and stereotypes must also be addressed in prevention interventions.

Finally, socioemotional competencies also play an important role. Deficiencies have been found in empathy, both in the cognitive and affective dimensions (Echeburúa et al., 2009, 2010; Boira and Tomas-Aragonones, 2011; Romero-Martínez et al., 2013, 2016; Echeburúa and Amor, 2016) as well as in the expression and understanding of the emotions of perpetrators of partner violence (Winters et al., 2004; Quinteros and Carbajosa, 2008; Boira, 2010; Boira et al., 2013). This deficiency is the root cause of the use of violence against romantic partners. In this sense, it has been suggested that the psychological construct of emotional intelligence can help clarify which mechanisms are needed to increase male awareness of intimate partner violence against women (Bosch and Ferrer, 2013). In addition, empathy has also been considered as a necessary skill for providing social support to victims (i.e., Sugarman and Hotaling, 1997; Weisz et al., 2007). Several authors likewise suggest that low self-esteem represents a risk factor for becoming a victim or perpetrator of intimate partner violence (Stith and Farley, 1993; Echeburúa and Fernández-Montalvo, 1997, 1998; Kesner et al., 1997; Sharpe and Taylor, 1999; Aumman, 2006; Santandreu et al., 2014).

Interestingly, addressing attitudes toward violence, gender roles and stereotypes, and socio-emotional competencies has been central to previous programs for the prevention of intimate partner violence in adolescence and youth. Programs addressing (1) attitudes toward partner violence can be seen in Jaffe et al. (1992), Lavoie et al. (1995), Avery-Leaf et al. (1997), Weisz and Black (2001), Schwartz et al. (2006), Alexander et al. (2014), Miller et al. (2014), Hines and Palm Reed (2015), McLeod et al. (2015), and Velasco (2015), as well as other programs developed by Díaz-Aguado and Martínez-Arias (2001), Hernando-Gómez (2007), Muñoz-Rivas (2010), Póo and Vizcarra (2011), and Fernández (2013); (2) gender stereotypes, sexist beliefs and attitudes and negative gender attitudes in adolescence and young adulthood in Foshee et al. (1998), Foshee et al. (2000), Foshee et al. (2004), Rainey and Rust (1999), Díaz-Aguado and Martínez-Arias (2001), Schwartz et al. (2006), Miller et al. (2014), and Velasco (2015); and (3) socioemotional competences such as self-esteem in Josephson and Proulx (1999) and Mateos-Inchaurrondo (2013), empathy in Hines and Palm Reed (2015), emotional intelligence in Murta et al. (2013), and emotional skills in Wolfe et al. (2009). Although all these programs have been implemented from adolescence onwards, the promotion of negative attitudes toward violence and gender inequality, and socioemotional learning are all aspects of paramount importance for children in primary education (Durlak, 1997; Durlak et al., 2010, 2011; Rimm-Kaufman and Hulleman, 2015).

Drawing on the above, this study is founded on the notion that intimate partner violence is multicausal as evidenced. As such, the theoretical framework is based on multicausal models (Riggs and O’Leary, 1989, 1996; Stith and Farley, 1993) and the integration of the ecological model (Ravazzola, 1997; Heise, 1998; Sluzki, 1998), the gender perspective (Olivares and Inchaustegui, 2011; White and Smith, 2011), the socio-emotional learning model (CASEL, Weissberg et al., 2015) and the multi-causal models (Riggs and O’Leary, 1989, 1996; Stith and Farley, 1993).

In view of the aforementioned, the main of objective was to design, implement, and evaluate a program for the prevention of gender-based intimate partner violence oriented at primary school children within the context of Colombia. Age was considered as a covariate due to the significant differences in cognitive development and knowledge acquisition among the different primary education courses (Fischer and Bullock, 1984). Gender was also taken into consideration given the existence of previous evidence pointing to gender differences in the flexibilization of gender stereotypes (Smetana, 1986; Henshaw et al., 1992; Signorella et al., 1993; Fernández-Rouco et al., 2019), the acceptance of aggression and the type of aggression used (Askew and Ross, 1991; Crick et al., 1997; Subirats and Tomé, 2007), empathy (Tobari, 2003; Mestre et al., 2009), self-esteem (Watkins et al., 1997; Israel and Ivanova, 2002; Frisén et al., 2014), and emotional intelligence (Bar-On and Parker, 2000; Karma and Maliha, 2005; Ferrando, 2006). As a result, consideration was given to the role of gender in the effectiveness of the program. In accordance with the main goal of this study, the following hypotheses were suggested:

(1) Controlling for the effect of age, participants in the experimental group will have lower scores in attitudes toward violence in partner relationships and with peers after the intervention compared to before the intervention, while participants in the control group will not show significant change.
(2) Controlling for the effect of age, participants in the experimental group will have lower scores in gender stereotypes after the intervention compared to before the intervention, while participants in the control group will not show significant change.
(3) Controlling for the effect of age, participants in the experimental group will have higher scores in socio-emotional skills after the intervention compared to before the intervention, while participants in the control group will not show significant change.
(4) Gender will play a moderating role in the effect of the intervention program on attitudes toward violence in partner relationships and with peers, gender stereotypes, and socio-emotional skills and attitudes.

## Materials and Methods

### Participants

The population sample included 344 children enrolled in the second and third grades of primary education at a Colombian public educational institution in the municipality of Chía, Cundinamarca. Of the sample, 56.7% of the participants were male and 43.4% were female. Participants ranged in age from 7 to 9 years, with an average age of 7.8 years (*SD* = 0.73). In relation to their socioeconomic level, 24.8% comprised the lower socioeconomic level, 68.7% the lower-middle, and 6.5% the middle level. In terms of family structure, 62.4% lived in a nuclear household (father, mother, and children living together); 11.7% included the extended family (in addition to the nuclear family, other relatives such as aunts, uncles, and grandparents lived in the household); 24.2% lived in a single-parent household in which the mother and her children lived together in the household; and 1.7% were part of a single-parent household in which the father and his children lived together in the household.

Regarding the parents’ occupations, the fathers had the following occupations: machinery operator (9.9%), public or private transportation driver (15.9%), company employee (24.6%), construction worker (7.5%), security guard (4.8%), retailer (19.2%), and other (18.1%). Mothers had the following occupations: machinery operator (12.2%), unpaid domestic worker (25.7%), company employee (34.6%), general services employee (9.3%), beauty and fashion specialist (1.5%), education provider (3.9%), and retailer (12.8%). The fathers’ education levels were as follows: 1.74% had received technical or professional training, while 98.20% held a high school diploma; and for mothers, 5.2% had received technical or professional training, while 94.8% held a high school diploma.

In addition, regarding their education level, the sample’s distribution was found to be 53.8% in second grade of primary school and 46.2% in third grade. Within each education level, students were distributed into groups, creating six groups in second grade and six groups in third grade. Further, 48.5% of the participants attended school in the morning and 51.5% in the afternoon.

The sample was distributed equally between the control and experimental groups, with 144 participants (41.9%) in the control group and 200 participants (58.1%) in the experimental group. The only significant difference found was for age [*t*(342) = 2,451; *p* = 0.015]; hence, this variable was controlled in the analysis of the results. On average, the experimental group was younger (Age = 7.7; *SD* = 0.73) than the control group (Age = 7.9; *SD* = 0.72).

### Procedure

This study was carried out in accordance with the recommendations of the following Colombian laws and protocols: which regulates the practice of the Psychology profession and dictates the code of ethics and bioethics on scientific, technical and administrative standards for health research (Resolution 8430 of 1993), in Law 379 of 1997 for research that includes statistics and data, in article 189 of the Political Constitution, in Law 1581 of 2012 and in Decree 1377 of 2013. All subjects gave written informed consent in accordance with the Declaration of Helsinki. Data was collected on two occasions, with a 3-month interim period. To begin collecting data, permission was requested from the educational institution to access its facilities and contact teachers and students. Subsequently, parents of students were asked permission for their children to participate in the intervention and to administer the tests using an informed consent form, which outlined the procedures, benefits, risks, and implications of their children’s participation in the program. Information was also provided concerning the protection of the identity of participants and persons involved in this context, ensuring confidentiality and the use of the information provided for statistical purposes only. Once parental permission was obtained, each of the primary school’s second and third grade classes was visited and each teacher was asked to allow some time to administer the tests.

Since the number of tests could cause fatigue in the participants, the tests were administered over a period of 2 weeks before the intervention and 2 weeks after the intervention with two sessions for each class on consecutive days. Each session lasted for an average of 1 h. The tests were administered in the classroom; each student was given a booklet with the questions and multiple choice options for each of the tests administered. During this period, participants were supervised to help resolve any questions that came up and to verify that the questionnaire was completed.

As access to classes for administering the tests and intervention depended on when the teachers could leave time in their class schedules, the control and experimental groups were distributed according to availability and scheduling, resulting in the aforementioned distribution: seven experimental groups (4 s grade and 3 third grade classes) and five control groups (2 s grade and 3 third grade classes).

The program lasted 3 months, with each experimental group receiving 2 1-h sessions every week. Each session was held in the classroom and the classroom teacher was present. During the first 10 min of each session, time was spent reviewing what was covered in the last session and the lessons learned, allowing children to share in their own words the activities completed and the lessons learned. Subsequently, we focused on the session’s core activities. At the end, the group drew conclusions on the topics that were covered.

The gender-based intimate partner violence prevention program covered three intervention units (gender, socio-emotional skills, and intimate partner violence), which were consistent with the predictors and intervening factors that were found in the theoretical and empirical review conducted. In turn, the program was designed by taking the characteristics of successful programs into consideration, such as taking guidance from rigorous models on gender-based intimate partner violence (ecological model, gender perspective, socio-emotional learning, and multi-causal models). In addition, successful psychoeducational principles such as experiential learning, *SAFE* (*sequenced, active, focused*, and *explicit*) skills training, socio-emotional learning (*SEL*), and the promotion of cognitive, emotional, and behavioral change were used for the intervention.

The program was divided into three didactic units: gender construction, gender-based partner violence, and socio-emotional skills. In general, this program aims to improve attitudes toward gender equality, decrease the acceptance of attitudes toward partner violence, and develop socio-emotional competencies as a means of preventing gender-based partner violence. Below (see Table 1) is a general overview of the contents and objectives of each unit.

**TABLE 1:** Summary of unit, objectives and contents of the program.

**Unit**	**Objective of the unit**	**Contents**
(1) Gender stereotypes	To make traditional gender roles and stereotypes more flexible by identifying them and recognizing the limitations they place on the unrestricted development of personality.	1.1. Gender precepts
		1.2. Professions and trades
		1.3. Domestic work
		1.4. Clothing, colors and toys
		1.5. Role problems
		1.6. The value of difference
		1.7. Construction of complete identities
		1.8. Importance of unrestricted personality development
		1.9. Promotion of gender equality from childhood onwards
(2) Gender-based intimate partner violence	Identify violence, its forms and implications, and recognize alternatives and solutions to gender-based intimate partner violence.	2.1. Recognizing violence
		2.2. Types of violence
		2.3. Alternatives and solutions to gender-based partner violence
(3) Socio-emotional competences	Develop the ability to recognize and value one’s own capabilities and emotions, identify the emotions of others and the importance of such, and regulate one’s own emotions and behaviors for non-violent conflict resolution.	3.1. Self-concept and self-esteem
		3.2. Emotional perception
		3.3. Emotional facilitation
		3.4. Emotional understanding and empathy
		3.5. Emotional regulation

### Measurement Scales: Variables

#### Attitudes Toward Intimate Partner and Peer Violence Attitudes Toward Violence in Partner Relationships

To assess the attitudes of acceptance toward violence in relationships, the Attitudes about Aggression in Dating Situations Scale (Slep et al., 2001; validated in Spanish by Muñoz-Rivas et al., 2011) was implemented. This scale assesses attitudes toward aggression in partner relationships by evaluating situations in which a man or a woman uses physical aggression against his or her partner. Participants have to state the degree to which they agree or disagree with the behavior being carried out by the person via a Likert-type scale with the following responses: “0” for “strongly disagree,” “1” for “somewhat disagree,” “2” for “somewhat agree,” and “3” for “strongly agree.” The scale contains 10 items, with a mean reliability in its original application of α = 0.70 (Slep et al., 2001), and α = 0.76, for the Spanish validation of the scale (Muñoz-Rivas et al., 2011).

The scale was assessed according to violence against women and men as well as justification for gender-based physical and verbal or psychological violence. Thus, this scale comprised of the following subscales: verbal violence (e.g., “Laura keeps laughing at Lorenzo in front of her friends, Lorenzo loses control and insults her”; α = 0.78 in the pre-test and α = 0.89 in the post-test), verbal violence against men (e.g., “Toni is teasing Rosa about her new haircut and tells her that it looks like a dog, Rosa gets very angry and yells at him.”; α = 0.69 in the pre-test and α = 0.79 in the post-test), verbal violence against women (e.g., “Miguel catches Carmen flirting with Roberto. Miguel gets very angry and yells at Roberto for flirting with Carmen”; α = 0.55 in the pre-test and α = 0.79 in the post-test), physical violence (e.g., “Miguel catches Carmen flirting with Roberto. Miguel gets very angry and hits Roberto for flirting with Carmen”; α = 0.82 in both the pre- and post-tests), physical violence against men (e.g., “Toni is teasing Rosa about her new haircut and tells her that it looks like a dog, Rosa gets very angry and pushes him”; α = 0.70 in the pre-test and α = 0.63 in the post-test), and physical violence against women (e.g., “Luis finds out Alicia’s been dating someone behind his back. He gets very angry and slaps her”; α = 0.66 in the pre-test and α = 0.77 in the post-test). This scale was measured using a Likert scale that evaluated the participants’ level of agreement with the proposed statements: “0” for “strongly disagree,” “1” for “somewhat disagree,” “2” for “somewhat agree,” and “3” for “strongly agree.”

#### Attitudes Toward Peer Aggression

To measure the acceptance of peer aggression, the Normative Beliefs about Aggression and Aggressive Behavior scale (Huesmann and Guerra, 1997) was used. This scale included 20 items that assessed primary school children’s normative beliefs about aggression by asking how positive or negative the participants felt about aggressive verbal or physical behavior against children. The scale comprised the following subscales: acceptance of weak provocation (e.g., “It’s wrong to take it out on others by saying cruel things when you’re angry”; α = 0.87 in both the pre- and post-tests for the acceptance of weak provocation), acceptance of strong provocation (e.g., “It’s generally okay to hit others when you’re angry.”; α = 0.92 in the pre-test and α = 0.91) acceptance of aggression against men (e.g., “Imagine a boy saying something mean to a girl. Do you think it would be wrong for the girl to yell at him?”; α = 0.83 in the pre-test and α = 0.82) and acceptance of aggression against women (e.g., “Imagine a girl saying something mean to a boy. Do you think it would be wrong for the boy to hit her?”; α = 0.94 in the pre-test and α = 0.91). The reliability in the original study was α = 0.80 for general acceptance of aggression, α = 0.75 for acceptance of weak provocation, α = 0.71 for acceptance of strong aggression, α = 0.70 for the acceptance of aggression against men, and α = 0.69 acceptance of aggression against women. Each item was measured using a Likert-type scale that assessed the participants’ level of agreement with the proposed statements: “0” for “strongly disagree,” “1” for “somewhat disagree,” “2” for “somewhat agree,” and “3” for “strongly agree.”

#### Gender Stereotypes

Gender stereotypes were measured using the Gender Stereotype Attitudes Scale for Children (Signorella and Liben, 1984). The original scale showed an average reliability of α = 0.88 among different samples (Signorella and Liben, 1985). It included 46 items divided into two scales: female gender stereotypes (e.g., “Who plays with dolls?”; α = 0.67 in the pre-test and α = 0.79 in the post-test) and male stereotypes (e.g., “Who plays with cars?”; α = 0.68 in the pre-test and α = 0.79 in the post-test). There are three response options: “males,” “females,” or “both.” Accordingly, for the scale with female stereotypes, the coding “0” was used for the response “females,” “1” for the response “males,” and “2” for the response “both.” For the scale with male stereotypes, “0” was coded as follows: “0” for the response “males,” “1” for the response “females,” and “2” for the response “both.” Thus, a higher score represented a less stereotyped attitude toward traditional gender roles. This process helped to clarify whether the child had a high or low level of gender stereotyping. In addition, using the same method as in the previous subscales, 10 items were added to analyze gender stereotypes in the context of a romantic relationship (e.g., “when you are dating someone, who gives gifts?, who pays the bill? who expresses his or her feelings?”). These items comprised the romantic relationships stereotypes subscale (α = 0.75 in the pre- test and α = 0.79 in the post-test).

#### Socio-Emotional Competences

Socio-emotional skills were measured using three variables: empathy, self-esteem, and emotional intelligence. The scales for each variable are detailed below.

##### Self-esteem

Self-esteem was measured using the Self-Esteem Scale (Rosenberg, 1965), which assesses personal self-esteem through questions on self-respect and feelings of self-worth. The reliability of this scale in the original study was found to be α = 0.80. It included 10 items (e.g., “I am convinced that I have good qualities”), although the inverse items or those that lowered the instrument’s reliability (items 2, 3, 5, and 10) were excluded in this study (α = 0.70 in the pre-test and α = 0.74 in the post-test). Each item was measured using a Likert scale that assessed the participants’ level of agreement with the proposed statements: “0” for “strongly disagree,” “1” for “somewhat disagree,” “2” for “somewhat agree,” and “3” for “strongly agree.”

##### Empathy

Empathy was measured using the Basic Empathy Scale (Sánchez-Pérez et al., 2014). It included 20 items that were divided into two subscales: one for affective empathy (e.g., “After being with a friend who is sad, for some reason I usually feel sad”; α = 0.66 in the pre-test and α = 0.63 in the post-test) and another for cognitive empathy (e.g., “I can understand my friend’s happiness when she or he does something well”; α = 0.63 in the pre-test and α = 0.67 in the post-test). Reliability coefficient of internal consistency in the original study was found as α = 0.73 for affective empathy and α = 0.63 for cognitive empathy (Oliva et al., 2011). Each item was measured using a Likert scale that assessed the participants’ level of agreement with the proposed statements: “0” for “strongly disagree,” “1” for “somewhat disagree,” “2” for “somewhat agree,” and “3” for “strongly agree.”

##### Emotional intelligence

Emotional intelligence was assessed using the Trait Meta-Mood Scale (TMMS) on Emotional States (from Salovey et al., 1995; validated in Spanish by Fernández-Berrocal et al., 1998; Fernández-Berrocal et al., 2004). It comprises three subscales: emotional support (e.g., “I am able to feel and express feelings appropriately”; Cronbach’s alpha = 0.70 in the pre-test and α = 0.78 in the post-test), emotional openness (e.g., “I understand my emotional states well”; α = 0.74 and α = 0.82 in the post-test), and emotional healing (e.g., “I am able to regulate my emotional states correctly”; α = 0.87 in the pre-test and α = 0.80 in the post-test). Each item was measured using a Likert scale that assessed the participants’ level of agreement with the proposed statements: “0” for “strongly disagree,” “1” for “somewhat disagree,” “2” for “somewhat agree,” and “3” for “strongly agree.” Each subscale presented good reliability with α > 0.80 in the original study (Fernández-Berrocal and Extremera, 2006).

### Statistical Analysis

The results were analyzed using the SPSS 22.0 statistical package which was used to provide a descriptive analysis of the averages, standard deviations, frequencies, and percentages used to characterize the population and each analyzed variable.

T-tests for independent samples and the chi-square test were performed for the differences in the distribution of the control and experimental groups in the socio-demographic variables (age, gender, socioeconomic level, and parents’ educational level). Furthermore, to analyze the program’s effectiveness, mixed ANOVAs with a within-subjects factor (when the group was measured), two between-subjects factors (group and gender), and a covariate (age) were used.

## Results

To measure the program’s effectiveness, 20 mixed ANOVAs with a within-subjects factor (when the group was measured), two between-subjects factors (group and gender), and a covariate (age) were used. The program was effective with regard to the following variables: male and female gender stereotypes, gender stereotypes in romantic relationships, normative beliefs regarding strong aggression, weak aggression, aggression against women and men among themselves, affective empathy, and attitudes about aggression in romantic relationships. In these cases, the time at which the interaction was measured was significant. The time at which group interaction by gender was measured was only significant with the dependant variable “acceptance of strong peer aggression” (*p* = 0.002) (see Table 2).

**TABLE 2:** Summary of the program’s effectiveness for the analyzed variables.

	**Pre**						** Post**									
	**Experimental group**			**Control group**			**Experimental group**			**Control group**						
				
													Time*Group		**Interaction**	
	**Male**	**Female**	**Total**	** Male**	**Female**	**Total**	**Male**	**Female**	**Total**	**Male**	**Female**	**Total**	**Interactions**		**interpretation**	
														
	**M**	**M**	**M**	**M**	**M**	**M**	**M**	**M**	**M**	**M**	**M**	**M**	***p***	***η*^2^ semi-**		
	***(SD)***	***(SD)***	***(SD)***	***(SD)***	***(SD)***	***(SD)***	***(SD)***	***(SD)***	***(SD)***	***(SD)***	***(SD)***	***(SD)***		**partial**	**Exp**	**Control**
**Attitude towards adressions (romantic relationships)**																
Verbal violence	1.03	0.92	0.98	0.83	0.77	0.81	0.76	0.83	0.79	0.81	0.73	0.77	0.167	0.007		
	(0.74)	(0.65)	(0.70)	(0.63)	(0.54)	(0.59)	(0.72)	(0.71)	(0.72)	(0.82)	(0.80)	(0.81)				
Verbal violence against men	1.03	0.93	0.99	0.71	0.61	0.67	0.78	0.81	0.79	0.73	0.65	0.69	0.076	0.011		
	(0.84)	(0.73)	(0.80)	(0.71)	(0.66)	(0.69)	(0.76)	(0.72)	0.74)	(0.80)	(0.74)	(0.77)				
Verbal violence against women	1.04	0.81	0.95	0.96	0.94	0.95	0.77	0.86	0.81	0.91	0.82	0.87	0.534	0.001	b > a*	
	(0.83)	(0.75)	(0.81)	(0.73)	(0.69)	(0.71)	(0.89)	(0.84)	(0.87)	(0.96)	(0.97)	(0.96)	0.017	0.020		
Physical violence	0.89	0.93	0.91	0.75	0.66	0.71	0.70	0.72	0.71	0.78	0.73	0.76	0.174	0.006	b > a**	
	(0.78)	(0.75)	(0.77)	(0.60)	(0.68)	(0.63)	(0.72)	(0.67)	(0.70)	(1.02)	(0.82)	(0.93)				
Physical violence against men	0.84	0.93	0.88	0.79	0.66	0.73	0.75	0.72	0.73	0.82	0.67	0.75	0.001	0.039		
	(0.82)	(0.85)	(0.83)	(0.75)	(0.73)	(0.74)	(0.76)	(0.72)	(0.74)	(1.41	(0.82)	(1.19)				
Physical violence against women	0.89	0.85	0.87	0.69	0.64	0.67	0.64	0.62	0.64	0.79	0.83	0.81	0.036	014		
	(0.90)	(0.82)	(0.87)	(0.68)	(0.72)	(0.70)	(0.76)	(0.72)	(0.74)	(0.90)	(0.95)	(0.91)				
**Attitudes toward aggression (peers)**																
Acceptance of weak aggression	0.42	0.33	0.38	0.58	0.44	0.52	0.36	0.20	0.29	0.79	0.44	0.63	0.015	019	b > a**	
	(0.72)	(0.64)	(0.69)	(1.81)	(0.69)	(1.10)	(0.58)	(0.44)	(0.53)	(0.96)	(0.64)	(0.84)				
Acceptance of strong aggression	0.44	0.26	0.36	0.56	0.52	0.54	0.23	0.26	0.24	1.02	0.47	0.77	0.16	0.18		
	(0.95)	(0.68)	(0.85)	(1.07)	(1.10)	(1.08)	(0.61)	(0.69)	(0.64)	(1.41)	(0.92)	(1.23)				
Acceptance of aggression against men	0.44	0.30	0.38	0.49	0.30	0.40	0.32	0.20	0.27	0.74	0.30	0.54	0.049	012		
	(0.78)	(0.70)	(0.74)	(0.73)	(0.57)	(0.66)	(0.56)	(0.46)	(0.52)	(0.98)	(0.54)	(0.84)				
Acceptance of aggression against women	0.39	0.32	0.36	0.70	0.69	0.70	0.36	0.22	0.30	1.00	0.67	0.85				
	(0.79)	(0.63)	(0.72)	(1.19)	(1.22)	(1.20	(0.64)	(0.49)	(0.59)	(1.18)	(1.06)	(1.14)				
**Gender stereotypes**																
Female stereotypes	1.02	0.96	0.99	0.93	0.91	0.92	0.75	0.78	1.00	0.99	0.000	0.95			b > a*** b < a*	
	(0.34)	(0.30)	(0.33)	(0.27)	(0.30)	(0.28)	(0.44)	(0.37)	(0.41)	(0.31)	(0.36)	(0.33)				
Male stereotypes	1.14	1.01	1.09	1.01	1.02	1.01	0.75	1.02	0.98	1.00	0.000	0.107			b > a***	
	(0.32)	(0.29)	(0.31)	(0.28)	(0.27)	(0.28)	(0.44)	(0.35)	(0.40)	(0.36)	(0.32)	(0.34)				
Stereotypes in romantic relationships	0.62	0.59	0.61	0.78	0.73	0.76	0.52	0.50	0.51	0.87	0.64	0.77	0.022	0.016	b> a** b < a*	
	(0.24)	(0.24)	(0.24)	(0.38)	(0.29)	(0.34)	(0.28)	(0.27)	(0.27)	(0.79)	(0.32)	(0.64)				
**Socio-emotional Skills**																
Self-esteem	1.84	1.95	1.88	1.66	1.87	1.76	1.77	1.74	1.76	1.54	1.67	1.61	0.774	0.000	b > a*	
	(0.74)	(0.65)	(0.68)	(0.77)	(0.69)	(0.74)	(0.71)	(0.69)	(0.70)	(0.71)	(0.78)	(0.74)				
**Empathy**																
Cognitive empathy	1.75	1.94	1.83	1.41	1.65	1.52	1.80	1.72	1.77	1.44	1.64	1.53	0.371	0.003		
	(0.65)	(0.60)	(0.63)	(0.60)	(0.59)	(0.61)	(0.69)	(0.56)	(0.64)	(0.64)	(0.61)	(0.63)				
Affective empathy	1.47	1.60	1.53	1.37	1.68	1.51	1.59	1.58	1.59	1.23	1.45	1.33	0.012	0.020	b > a**	
	(0.73)	(0.73)	(0.73)	(0.75)	(0.64)	(0.71)	(0.73)	(0.63)	(0.68)	(0.59)	(0.70)	(0.65)				
**Perceived Emotional Intelligence**																
Emotional support	1.62	1.85	1.72	1.59	1.81	1.69	1.60	1.70	1.65	1.47	1.68	1.57	0.693	0.001		
	(0.62)	(0.57)	(0.61)	(0.72)	(0.58)	(0.67)	(0.72)	(0.55)	(0.65)	(0.76)	(0.72)	(0.75)				
Emotional clarity	1.65	1.86	1.74	1.48	1.53	1.50	1.64	1.76	1.69	1.35	1.53	1.43	0.879	0.000		
	(0.72)	(0.57)	(0.66)	(0.71)	(0.56)	(0.64)	(0.74)	(0.58)	(0.68)	(0.80)	(0.74)	(0.78)				
Emotional healing	1.81	1.89	1.85	1.38	1.44	1.40	1.78	1.87	1.81	1.36	1.45	1.40	0.847	0.000		
	(0.70)	(0.54)	(0.63)	(0.64)	(0.64)	(0.64)	(0.66)	(0.50)	(0.60)	(0.81)	(0.72)	(0.77)				

*b: before; a: after. ^∗^*p*-Value < 0.05; ^∗∗^*p* < 0.01; ^∗∗∗^*p* < 0.001 (in this case, for the *p*-value of the *post hoc* multiple comparison test with the Bonferroni correction).*

The significant interactions are described below.

**FIGURE 1:**
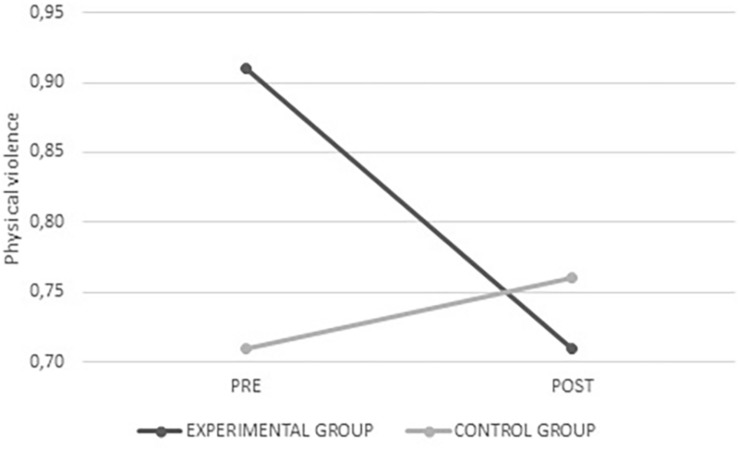
Average values for physical violence from the experimental and control groups before and after intervention.

**FIGURE 2:**
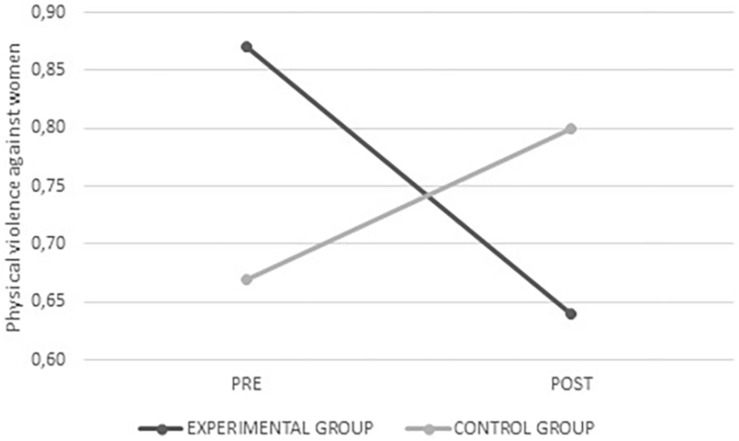
Average values for physical violence against women from the experimental and control groups before and after intervention.

Significant interactions were found with the physical violence (*F*_(__1_,_284__)_ = 5.579; *p* = 0.017) and physical violence against women (*F*_(__1_,_283__)_ = 11.483; *p* = 0.001) variables. In these cases, the experimental group had lower scores, namely, less justification of aggression in romantic relationships after intervention (see Figures 1, 2). No significant interactions were found for the remaining variables in this scale (physical violence against men, psychological violence, psychological violence against men, and psychological violence against women); however, there was a tendency toward lower levels of justification for aggression in romantic relationships in the experimental group.

**FIGURE 3:**
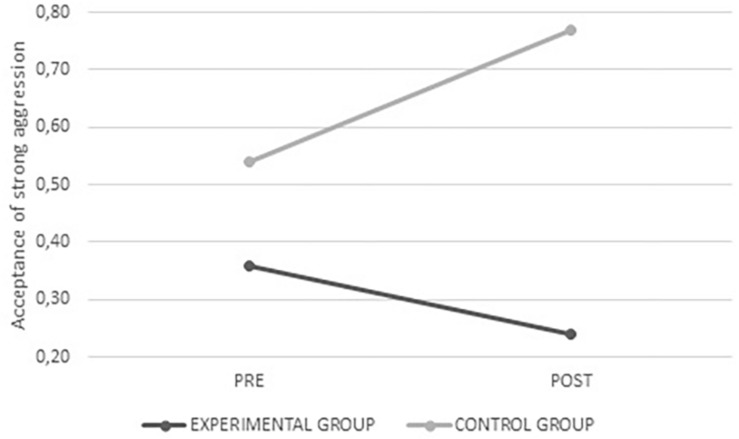
Average values for acceptance of strong aggression from the experimental and control groups before and after intervention.

**FIGURE 4:**
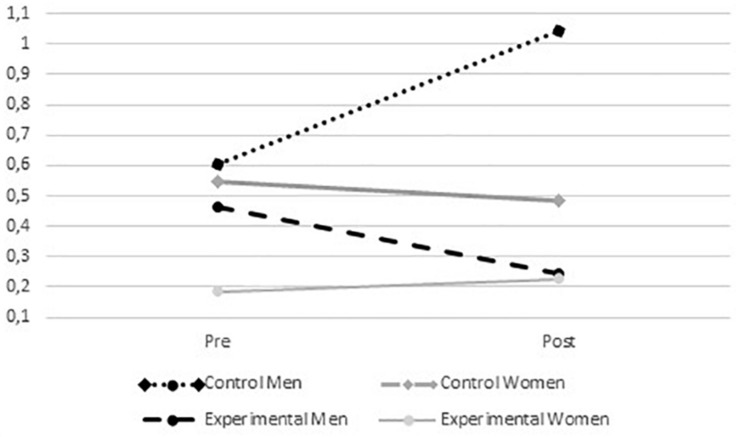
Average values in acceptance of strong aggression from the experimental and control groups according to gender before and after intervention.

In relation to normative beliefs on peer aggression, for the acceptance of strong aggression, a significant interaction was found among the time of measurement^∗^group^∗^gender (*F*_(__1_,_315__)_ = 11.417; *p* = 0.001). In this case, it was possible to identify that the male participants in the control group had an increased average value after intervention, in contrast to the male participants in the experimental group, who showed decreased scores. No differences were found for female participants in the experimental and control group prior to and after the intervention (see Figures 3, 4). These gender differences suggest that the program was more effective for men regarding this variable.

**FIGURE 5:**
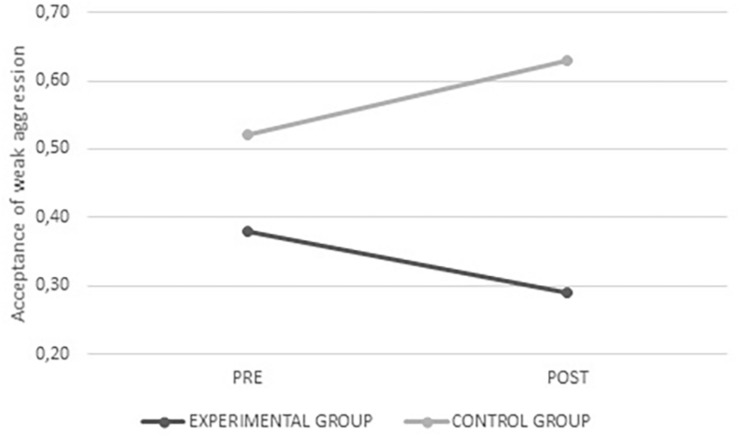
Average values for acceptance of weak aggression from the experimental and control groups before and after intervention.

**FIGURE 6:**
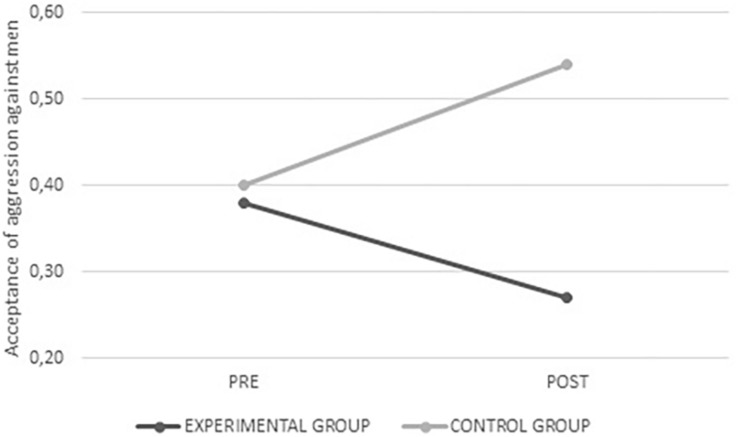
Average values for acceptance of aggression against men from the experimental and control groups before and after intervention.

**FIGURE 7:**
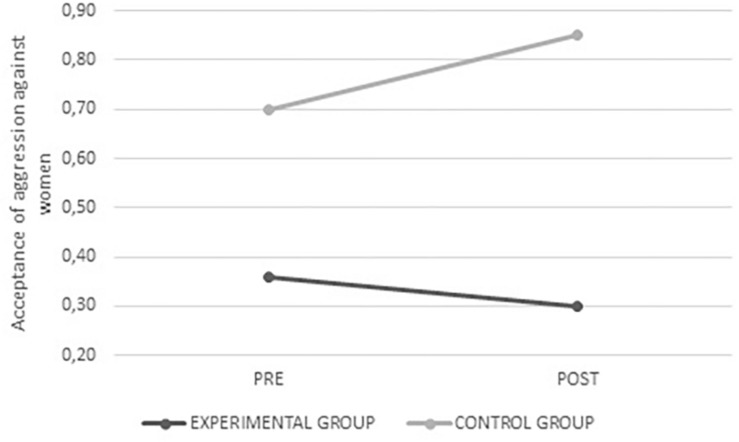
Average values for acceptance of aggression against women from the experimental and control groups before and after intervention.

In addition, significant interactions were found for the acceptance of weak aggression (*F*_(__1_,_320__)_ = 4.426; *p* = 0.036), acceptance of aggression against men (*F*_(__1_,_320__)_ = 5.899; *p* = 0.016), and the acceptance of aggression against women (*F*_(__1_,_337__)_ = 3.882; *p* = 0.049). It was found that the average values decreased after intervention exclusively in the experimental group, showing less justification for the use of peer aggression after the program was administered (see Figures 5–7).

**FIGURE 8:**
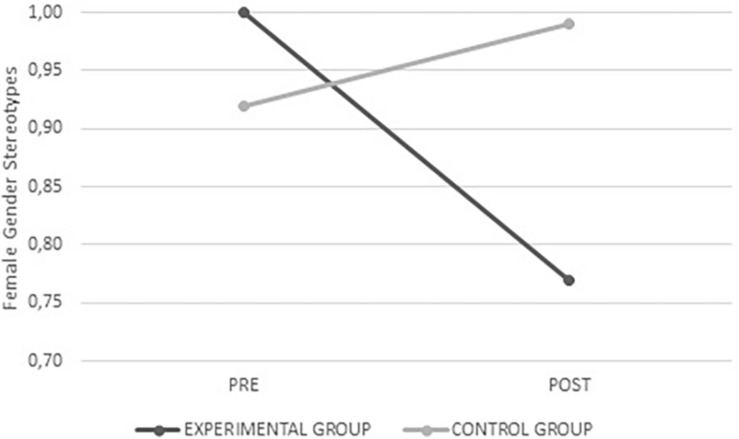
Average values for female gender stereotypes from the experimental and control groups before and after intervention.

**FIGURE 9:**
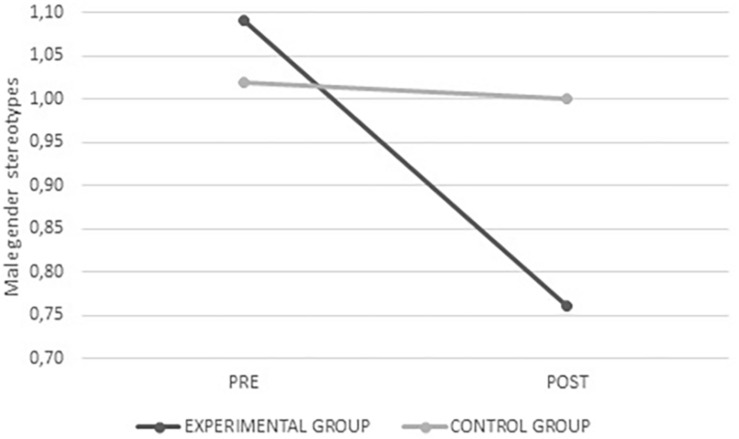
Average values for male gender stereotypes from the experimental and control groups before and after intervention.

**FIGURE 10:**
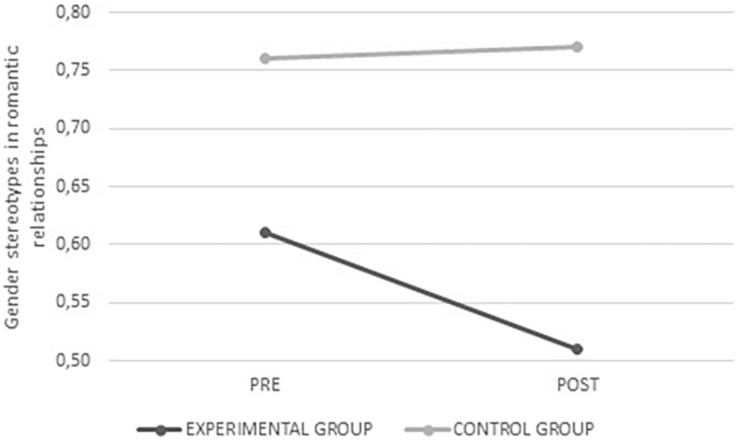
Average values for gender stereotypes in romantic relationships from the experimental and control groups before and after intervention.

Female gender stereotypes *F*_(__1_,_334__)_ = 34.984, *p* < 0.001, male gender stereotypes *F*_(__1_,_334__)_ = 39.995, *p* < 0.001, and those specific to romantic relationships *F*_(__1_,_326__)_ = 5.296, *p* = 0.022 scored lower after the intervention in the experimental group, whereas no change was observed in the control group. In all three cases there were fewer stereotyped beliefs after the intervention (see Figures 8–10).

**FIGURE 11:**
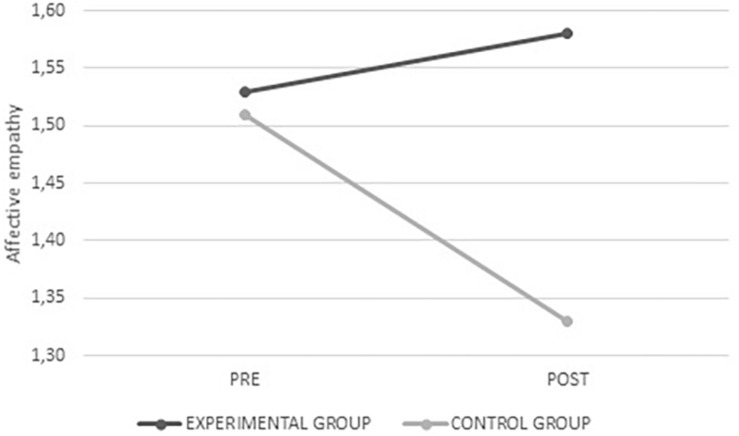
Average values for affective empathy from the experimental and control groups before and after intervention.

Finally, for empathy, significant interaction with affective empathy was observed, wherein a stable average was identified in the experimental group after intervention, in contrast to a lower score in the control group (*F*_(__1_,_311__)_ = 6.362; *p* = 0.020) (see Figure 11).

No age effect (covariate) was observed in any of the analyses.

## Discussion

### Attitudes Toward Violence in Intimate Partner Relationships

The program was effective for the variables of general physical violence and physical violence against women, as the participants of the experimental group reduced their mean approval in these variables. When comparing these findings to those obtained in other programs for the prevention of partner violence in youth and adolescents, several programs were identified that have also reduced the mean in relation to attitudes and beliefs that justify partner violence in their experimental groups (i.e., Muñoz-Rivas, 2010; Póo and Vizcarra, 2011; Fernández, 2013; Hines and Palm Reed, 2015; McLeod et al., 2015; Velasco, 2015).

It is important to note, however, that in the analysis of the scales used, most of these programs made no distinction between attitudes regarding physical and verbal violence or the gender of the victim. The only program that analyzed forms of aggression was that of Muñoz-Rivas (2010), who found that attitudes justifying physical violence were reduced and extended over time in contrast to decreases in attitudes justifying psychological violence, which once again increased in the follow-up assessment. This finding, compared to the conclusions of this study, may be a hindrance to bringing about significant changes with respect to the devaluation of verbal violence in couples, although this hypothesis needs to be studied further.

Conversely, in terms of the results of the program’s effectiveness on general physical violence and that against women, comparisons with other studies are challenging as there is no existing research that measures attitudes toward aggression in middle childhood relationships. These findings can nonetheless be interpreted by considering other studies on the development of aggression during childhood and the findings of other investigations on the meaning of violence in childhood, which may shed more light on the findings of this study.

Studies on childhood aggression have shown that in middle childhood, verbal abuse is more common than physical aggression. Further, the importance given to the social sphere (peers and teachers, among others) causes children to seek new cooperative ways to solve conflicts, as their understanding of the rules of behavior as agreements made for the common good increases, leading the child to judge behavior according to its consequences (Furman, 1982; Coie et al., 1991; Hartup, 1992; Newcomb and Bagwell, 1995; Hartup and Stevens, 1999; Pellegrini and Archer, 2005; Delgado and Contreras, 2009; Etxebarria, 2014; Swit et al., 2016). We therefore speculate that the participants of this study may have applied the socioemotional learning acquired during the program to the assessment of situations of partner violence.

This can be reinforced if one considers the findings of Piedrahita et al. (2007) on the meaning of violence in childhood. Their study showed that Colombian children between the ages of 6 and 8 recognize a situation as being either good or bad and therefore consider fighting to be an inadequate solution to conflict, with negative consequences for all involved. In relation to partner violence, they express displeasure when it comes to fights among their parents or when their father strikes their mother. The children consider that having seen this behavior in their parents could lead them to reproduce it themselves. Finally, they state that the family should seek solutions and maintain loving relationships.

Furthermore, the UN Latin America Regional Commission (2005) conducted a study with 1,800 children and adolescents in 17 Latin American countries and found that with regard to partner violence among their parents, the children showed displeasure; the children considered partner violence to be a form of violence toward them as well and believed that these fights had an affect on their academic performance. In the Spanish context, a study carried out by Bello (2016) with children aged 10–12 years old showed that they considered the violence they witnessed to be of a reproductive nature, as they believed that in relation to the violence that occurred between their parents, for example, in the cases that this violence occurred in front of them, they could imagine themselves replicating it. Furthermore, regarding abuse against a person of the opposite sex, children affirm the importance of reporting these cases of abuse and consider it important that in order to prevent such cases, people – adults, children and society in general – should be made aware of and educated accordingly.

Based on the aforementioned information, it can be identified that, in general and in accordance with the results of this research, children from different contexts tend to disapprove of violence. Furthermore, it is important to note that although the implications of the findings in these studies relate to either generalized violence or violence among the parents of the children who participated in the study, the findings nevertheless provide some insight on how children interpret situations of violence in the context of romantic relationships.

The fact, however, that the program results were effective for the variables on physical violence and physical violence against women confirms the greater tendency in middle childhood to accept and express subtle, or verbal aggressions. This compares with the approval and use of harsher forms, such as physical aggression; in addition, it indicates that violence against men continues to be considered more acceptable than violence against women in the context of intimate relationships. Consequently, the association of attributes such as strength and resilience ascribed to men persists, despite the significant decrease of male gender stereotypes. This may be explained by the more static and rigid perception of boys and girls with respect to male stereotypes (Smetana, 1986), or it may indicate the presence of spontaneous stereotyping when assessing this type of situation.

By contrast, there was a greater disapproval of physical violence against women, which may be due to the fact that they are considered more vulnerable to this type of abuse. This may be the result of the gender stereotyping of women’s weaknesses and sensitivities. However, owing to the fact that the level of approval decreased significantly throughout the intervention and the levels of female gender stereotypes were reduced, this may indicate that the program allowed for a more realistic observation on the forms of partner abuse and, via these activities, women were recognized as the group most frequently affected by partner violence.

### Attitudes Toward Aggression Among Peers

In turn, the intervention was effective in influencing attitudes toward aggression among peers, since it was observed that the experimental group showed lower levels of approval than the control group in the variables of soft and strong aggression against women and men after the intervention.

With respect to the effective results of the program concerning the acceptance of all forms of aggression assessed, it may be stated that the results are in accordance with the developmental stage of the participants, as several authors have reported that children from 6 to12 years of age tend to reduce physical aggression to make way for more subtle forms of aggression (Furman, 1982; Hartup, 1992; Newcomb and Bagwell, 1995; Coie and Dodge, 1998; Hartup and Stevens, 1999; Pellegrini and Archer, 2005; Delgado and Contreras, 2009; Etxebarria, 2014; Swit et al., 2016). The experimental group, however, also showed a significant decrease in the approval of soft or verbal aggression, which would mean that the program achieved significant changes in all forms of aggression acceptance, beyond the effects at the developmental stage.

In the strong aggression approval variable however, it was found that after the intervention, the program was effective only for boys, and not for girls. In general terms, previous studies have indicated that the reported approval of aggression in children is often mediated by social approval and normative gender beliefs (Martin et al., 2002; Giles and Heyman, 2005; Smith et al., 2009). In addition, it has been found that boys tend to approve of aggression at higher rates than girls (Huesmann and Guerra, 1997), and that they rely more heavily on physical aggression than girls (Archer, 2004; Côté et al., 2006). As a result, the program could have had a differential effect owing to the fact that the approval of strong violence is more prevalent in boys and, furthermore, the girls in the experimental group had very low levels of approval of violence before the intervention, making it virtually impossible to decrease their approval after the intervention. Only in this case was hypothesis 4 confirmed.

Finally, the changes made in the acceptance of peer aggression variables are fundamental in preventing gender-based intimate partner violence since, as stated by Underwood and Rosen (2009), “gender differences in relationships among peers in middle childhood may set the stage for difficulties in emerging heterosexual romantic relationships in adolescence” (p. 4). Therefore, given the relationship between the attitudes toward the acceptance of aggression and aggressive behavior (Huesmann et al., 1992; Tolan et al., 1995; Sprinkle, 2010; Busching and Krahé, 2015), a decrease in the acceptance of aggression in boys and girls has a positive effect when it comes to relating with peers and may lead to healthier future romantic relationships.

### Gender Stereotypes

The program was effective in the gender stereotypes variable since the experimental group showed significantly lower levels of male and female gender stereotypes and relationships after the intervention, whereas members of the control group either significantly increased their level of stereotype (female and in relationships) or remained stable (male stereotypes). Thus, the second hypothesis of this study is proven.

Similar results were found in other programs that aimed to prevent intimate partner violence, given that these programs also led to a decrease in gender stereotypes (Foshee et al., 1998, 2000, 2004), sexist attitudes and beliefs (Díaz-Aguado and Martínez-Arias, 2001; Schwartz et al., 2006; Velasco, 2015), and in negative attitudes toward gender equity (Miller et al., 2014). However, all the aforementioned programs were carried out with youth and adolescents, making a comparison with respect to age difficult. With respect to the programs focused on decreasing gender stereotypes in childhood, the “Words can Hurt You” anti-bias curriculum program by Rainey and Rust (1999) was aimed at middle childhood boys and girls, and significant differences in the experimental group were found after the intervention, as boys and girls within this group presented an increased number of androgynous answers measured through the Gender-Stereotyped Attitude Scale for Children, showing greater flexibility of gender stereotypes.

Having discussed other programs, it is important to explain the results of the program’s effectiveness on gender stereotypes in further detail. First, the participants’ age (7-9 years old) corresponds to the period of gender flexibility, which makes it possible to reduce the level of stereotypes (Freixas Farré, 2012). However, in addition to flexibility, knowledge on gender stereotypes remain stable during this stage (Huston, 1983, 1985; Ruble et al., 2006), and it has been shown that flexibility alone is not enough to reduce spontaneous stereotyping, although it may be influenced by knowledge of stereotypes (Devine, 1989; Strack and Deutsch, 2004). This is noteworthy since it can be understood that the content discussed with the participants in the experimental group led to a decrease in gender stereotyping levels, despite the application of the program at an age that facilitated the receptivity of participants’ concepts about non-traditional genders. Such changes were not observed in the control group participants.

Before ending this section, it is important to mention that positive results of the intervention on gender stereotypes and attitudes toward violence can have a cumulative effect since previous research has shown a clear relationship between sexism and acceptance of gender-based violence (Valor-Segura et al., 2014; Fernández-Fuertes et al., 2018).

### Socio-Emotional Skills

#### Empathy

The results on the effectiveness of the program regarding the socio-emotional skills assessed (self-esteem, empathy, and emotional intelligence) demonstrate significant differences on the emotional empathy variable in the experimental group. In this sense, the experimental group maintained the same levels of empathy while the control group presented a significant drop. Therefore, the program had a protective effect on empathy in the experimental group.

After analyzing the result obtained on the effectiveness of maintaining emotional empathy but not cognitive empathy, it may be stated that, according to the conceptualization made by Preston and de Waal (2002) on empathy components, the participants in the experimental group provided more emotionally charged responses to other people’s emotions (emotional empathy), though no changes were detected in comprehension and/or understanding of other people’s feelings (cognitive empathy). It could be said that these findings confirm that emotional and cognitive empathy develop separately and follow different paths (Hodges and Klein, 2001; Decety and Jackson, 2004; Eisenberg and Eggum, 2009), although cognitive processes are necessary in the emotional comprehension that preceds emotional processes (Fernández-Pinto et al., 2008).

This would imply that, based on the scale punctuations, mid-to-low levels of cognitive empathy could be sufficient to stimulate emotional empathy stability. It is important to note that the mean emotional empathy prior to the intervention was lower than the mean cognitive empathy and, after the intervention, although the emotional empathy score was maintained in the experimental group, cognitive empathy continued to have a higher mean than emotional empathy in the group. Therefore, although this difference in mean is quite small, it can be concluded that the program achieved a balance between the levels of cognitive and emotional empathy.

Finally, the aforementioned details show that the applied and assessed program had a positive impact on empathy, indicating the need to continue investigating the impact of interventions for the prevention of intimate partner violence through empathy at different ages, as well as a simultaneous examination of the effects of its components.

#### Self-Esteem

The program was not effective on the self-esteem variable given that the experimental group presented no significant changes with the intervention. This can be explained by some contextual elements which may have caused a negative effect on self-esteem. In general terms, it was possible to identify that the children who participated in this study were exposed to constant criticism from their teachers, even in sessions aimed at building self-esteem, leading to contradictory messages for the students. Furthermore, the sessions conducted for this research were constantly interrupted by school activities, and on several occasions the teachers interrupted the sessions to begin their classes. This situation prevented the programed activities from being carried out as initially planned. Also in middle childhood, social norms and rules significantly affect self-concept and increase negative feedback from teachers, leading to low self-esteem (Robins and Trzesniewski, 2005). Research has shown that students’ feelings of satisfaction and comfort with their teachers influence self-esteem (Dessel et al., 2017), that the way in which teachers deal with situations affect the students’ self-image (Bruno and Njoku, 2014), and that negligence by teachers may bring about low self-esteem (Obidigbo, 2006). Moreover, the importance of feedback from teachers through compliments or criticism has been studied, finding that compliments and positive statements to their students have a positive impact on their self-esteem, self-concept, positive self-talk, and on the classroom environment as a whole (Burnett, 1996, 2003; Yaratan and Yucesoylu, 2010).

Another plausible explanation might be that the measure of self-esteem is quite broad and readily susceptible to maximum social desirability if applied in a group setting, even though the conditions of anonymity and confidentiality were maintained.

Nonetheless, in spite of the limitations encountered, it is important to remember that the level of self-esteem in the experimental groups tended to be more stable. This may be considered a contributing factor as the program, although not significant, reveals the potential for obtaining greater effects by allowing for the time and availability needed to implement the self-esteem sessions.

#### Emotional Intelligence

The program was ineffective with regard to emotional intelligence, since no significant differences were found on attention, clarity, and emotional redress in the experimental group after the intervention. Thus, it is important to analyze these results in comparison to other interventions aimed at preventing partner violence as well as at improving emotional intelligence.

Regarding programs on prevention of partner violence, none of the ones analyzed here in assessed the emotional intelligence level, although two programs assessed some of its components: the Murta et al. (2013) program evaluated the difficulties to regulate emotions. Nevertheless, after the intervention, the quantitative assessment of this variable showed no significant changes, although students showed an increase in the use of emotional expressions and social skills at the qualitative level. Furthermore, the Fourth “R” program by Wolfe et al. (2009) assessed the emotional stress and found that the experimental group participants reported less emotional abuse by their partners after the intervention.

It is important to note that studies on emotional intelligence in childhood that assess the three components of emotional intelligence through the TMMS scale used in this study are usually of the correlational type. This makes it difficult to identify the attention, clarity, and emotional redress levels obtained from the samples of the children assessed. Thus, for example, studies that seek to determine the influence of parental styles on the emotional intelligence of their children (Baumrind, 1978; Lopes et al., 2004; Bennett et al., 2005; Páez et al., 2006; Schaffer et al., 2009; Asghari and Besharat, 2011; Sánchez-Núñez et al., 2013; Stein et al., 2013; Ramírez-Lucas et al., 2015) and studies that associate childhood emotional intelligence with other variables such as social competence (Rockhill and Greener, 1999), social achievement (Fatum, 2008), and academic adjustment to school (Mestre et al., 2006) can be found. However, this makes it difficult to compare the results found in this study to those of other studies.

In accordance with the above, and given that the program assessed herein included several of the characteristics of effective programs (objectives and clear goals, assessment severity, work on developing skills, use of experiential activities), one differentiating element might have been the focus on working with other actors within the environment such as fathers, mothers, and teachers. As stated earlier, the school environment created limitations that may be settled via complementary education with respect to emotional skills. In addition, a longer intervention program that includes a more substantial contribution from the context (namely, parents and teachers) could have resulted in significant changes to emotional intelligence.

### Limitations of the Study and Future Lines of Research

The main limitation of this study was that communication with the teachers and families was not effective, though the intervention with the children was. Group management was difficult because some teachers interrupted sessions of intervention and evaluation or gave conflicting messages regarding the contents of the program. With regard to the family, greater inclusion in the program activities would have been relevant, however due to the socio-economic and working situation of the parents, it was not possible for them to be actively involved. All of the above highlights the need for intervention with parents and teachers, not only to improve the effects of the program on participating children, but also to promote coherence between the principles of the program and the beliefs and attitudes of the community. These effects were designed to facilitate the development of socio-emotional skills and to increase knowledge for the promotion of healthy relationships and general well-being in family and school contexts. All these aspects could have a greater impact on the prevention of gender-based dating violence.

Another element that may have limited the research results was the type of instruments used for evaluation, given that self-reports were used for program evaluation. Although this technique is used in other programs, it can generate reports based on the social desirability, particularly for scales that evaluate aspects related to social norms (Chaux, 2003). However, according to the results, it was possible to ascertain that the students responded sincerely. To reduce bias due to social desirability, participants were reminded throughout the administration of the tests that there were no right or wrong answers. They were also told that their response was anonymous.

Another aspect to consider is that the effects of the program were not monitored 3 or 6 months after its application. A follow-up evaluation would have been useful in identifying the effects of the program in the medium term. In addition, it is equally relevant to note that the effects of the program directly on gender-based intimate partner violent behavior are unknown. Subsequent measurements of the effectiveness of the program over time are necessary owing to the fact that children at this age are often not in couple relationships, even if they have knowledge about them. However, this intervention achieved significant effects on most of the factors involved in its development. It would therefore be important to continue and expand the work of this research by testing the same participants at different periods of their lives, while meanwhile replicating the program at different stages of the life cycle (i.e., adolescents).

Lastly, according to the aforementioned limitations, the following elements have been foreseen as worth consideration for future investigations: (1) development of gender-based violence prevention programs that include training for teachers and parents of the participating students; (2) implementation of programs in the families and in the community regarding the prevention of gender-based partner violence as a means of generating more coherent changes with the dynamics of the context; (3) use of different types of assessment instruments (quantitative and qualitative) and different sources of information on the changes made (parents, teachers, teachers and other caregivers); (4) given the importance of a cross-cultural perspective to understand the effects of each culture and children’s learning environments on development (Best and Williams, 1993), it would be pertinent to compare the results obtained in this research with children of other countries, which would also allow the effects of the program to be gauged depending on the context of application; (5) carrying out measurements of the effectiveness of the program by reapplying the tests to the children participating in this research during adolescence, in order to monitor its effects; (6) conducting research and longitudinal interventions that allow primary and secondary prevention of gender-based dating violence in childhood and adolescence, thereby evaluating the effectiveness of the programs over time.

## Conclusion

Overall, the program presented adequate levels of effectiveness that can be improved upon with a larger degree of control and support from the school environment. This allows for the possibility of replicating the program within other contexts and populations with the primary aim of strengthening its effects to prevent gender-based intimate partner violence. Furthermore, the level of innovation of this program gives rise to a new line of research on the prevention of gender-based partner violence from childhood.

Given that the Colombian context faces particular conditions (high rates of gender-based partner violence, poor education on gender equality, and deficiencies in socio-emotional skills), these factors indicate the need to apply the suggested program, taking into account the implications of its effectiveness, in order to reduce the risk factors entailed in gender-based partner violence. Thus, in light of the lack of these types of programs and, moreover, the lack of effective programs in Colombia, the importance of continuing and expanding psychological research in the Colombian context has been highlighted so as to promote personal and social well-being as well as to prevent gender-based intimate partner violence in primary education.

## Data Availability Statement

The datasets generated for this study are available on request to the corresponding author.

## Ethics Statement

Ethical review and approval was not required for the study on human participants in accordance with the local legislation and institutional requirements. Written informed consent to participate in this study was provided by the participants’ legal guardian/next of kin in accordance with the local legislation and institutional requirements for the study on human participants.

## Author Contributions

AG and RC conceived and designed the study, wrote the sections of the manuscript, revised the manuscript, and read and approved the submitted version. AG organized the database and wrote the first draft of the manuscript. RC performed the statistical analysis.

## Conflict of Interest

The authors declare that the research was conducted in the absence of any commercial or financial relationships that could be construed as a potential conflict of interest.
